# The Relationship Between Uterine Artery two-dimensional Color Doppler Measurement and Pregnancy Outcome: A Prospective Observational Study

**Published:** 2017

**Authors:** Sudha Prasad, Ritu Goyal, Yogesh Kumar, Poonam Nayar, Supriya Hajela, Aswathy Kumaran, Rajesh Vairagi, Shalini Chauhan

**Affiliations:** 1- Department of IVF and Reproductive Medicine, Maulana Azad Medical College & Associated Lok Nayak Hospitals, New Delhi, India; 2- Mata Chanan Devi Hospital, New Delhi, India

**Keywords:** Doppler ultrasound, Follicular phase, IVF-ET, Pregnancy outcomes, Uterine artery

## Abstract

**Background:**

The purpose of the study was to evaluate the role of uterine blood flow parameters measured by uterine artery two-dimensional (2D)-power color doppler (PCD) ultrasound in predicting fertility outcomes in women undergoing IVF-ET cycles.

**Methods:**

In this prospective observational study, a total of 188 infertile women who underwent IVF-ET cycles were investigated. Uterine artery 2D-PD measurements were taken during early follicular phase and on day of trigger. Pulsatility Index (PI), Resistant Index (RI), Peak Systolic Velocity (PSV), and Systolic/Diastolic ratio (S/D) were measured. Statistical correlation was sought between the doppler parameters and fertility outcomes.

**Results:**

The pregnancy rate was 40.43% (76/188). The women who conceived (n= 76) (Group A) were found to have mean age of 31.2±3.9 years whereas the non-pregnant group of women (n=112) (Group B) had mean age of 31.45±4.25 years. The mean PI measurements subsequently during early follicular phase and on the day of hCG trigger between group A and group B were comparable (2.09±1.15 versus 1.9±0.95; p=0.385 and 1.86±1.12 versus 2.03±1.0; p=0.192, respectively). No significant changes in the uterine artery PSV values and S/D values and RI were noted during the cycle.

**Conclusion:**

Uterine artery doppler evaluation in women undergoing IVF cycles was not predictive of the pregnancy outcomes.

## Introduction

Infertility is a deeply worrying experience, physically and psychologically. There are evidences that increased stress is associated with poorer pregnancy outcome in women undergoing assisted reproduction cycle. Substantiation from obstetrics studies has shown that increased maternal distress can lead to abnormal uterine and placental perfusion responsible for poor pregnancy rates.

The World Health Organization (WHO) estimates that approximately 8%–10% of couples experience infertility problem due to various explained and unexplained reasons. *In vitro* fertilization and embryo transfer (IVF-ET) is endorsed and is selectively and readily available for the management of infertility. The most exasperating problem with the IVF-ET is implantation failure, which decreases the treatment success rate to 15–45% (1). The positive pregnancy outcome depends upon diverse aspects related to IVF cycles. Uterine perfusion and its impedance have been found to be an indicator for the likelihood of subsequent implantation (2–4).

Abnormal uterine artery velocimetry is associated with unexplained sub-fertility, poorer uterine receptivity to implantation, and recurrent miscarriages (5, 6).

Uterine blood flow is an important factor contributing to uterine receptivity (7) and can be studied by means of two-dimensional (2D)-power color doppler (PCD) ultrasound (8, 9). Utilizing this technique, it was feasible for some authors to distinguish between conception and non-conception cycles of women prior to going through IVF-ET cycle (6, 10).

Based on the above evidence, this study was conducted to address the role of 2D-PCD ultrasound performed prior to embryo transfer procedure in IVF-ET program. The findings were measured and analyzed between conception and non-conception IVF cycles of these women. The predictive role of doppler velocimetry was based on the differences found in mean uterine artery pulsatility index (PI), resistance index (RI) and peak systolic velocity (PSV), systolic/diastolic velocity ratio (S/D) values between pregnant and non-pregnant groups of women.

## Methods

The present study was conducted at IVF & Reproductive Biology Centre, Department of Obstetrics and Gynecology, Maulana Azad Medical College and associated Lok Nayak Hospital, New Delhi. Women who attended Fertility and IVF Clinic for their infertility management were enrolled. A total of 188 cycles of the age-matched women who consecutively underwent IVF/ICSI-ET procedure were investigated to assess the fertility outcome based on 2-D-PCD measurements.

Ethical clearance was obtained from Institution Ethics Committee (IEC) of the college and associated hospitals. Selection of the women was carried out on the basis of following inclusion and exclusion criteria:

**Inclusion criteria**
1. Infertile women having the age ≤38 years.
2. Baseline (Day2/3) FSH and LH levels ≤10 *mIU/ml*
3. Baseline (Day2/3) Estradiol (E_2_) levels ≤50 *pg/ml*
4. Normal Serum Prolactin levels <20 *ng/ml*
5. TSH levels <3.5 *mIU/ml*

**Exclusion criteria**
1. Women having infertility attributed to endocrinal abnormalities.
2. Previously documented poor response to ovarian stimulation.
3. Premature ovarian failure.
4. Stage III and IV endometriosis.
5. Known psychological disorders.
6. Uterine anomalies.
7. Uncompensated heart diseases.
8. Inadequate response of endometrial lining (endometrial thickness <7 *mm*)
9. Moderate to severe OATS

All patients were managed by tailored gonadotropin stimulation protocol. The ovarian stimulation was achieved by long agonist protocol or antagonist protocol. A TVS scan was performed to assess the antral follicle count (AFC) and endometrial thickness (ET) on day 2/day 3 and to rule out any persisting ovarian cyst. Serum FSH, LH, E2, PRL, serum progesterone (P4) and TSH levels were also measured prior to starting ovarian stimulation. Approximately, 2.0 *ml* of blood sample was withdrawn from ante-cubital vein and send to radio immune-assay (RIA; RIA MAS analyzer 12 well gamma counter, Stratec Biomedical System AG Gewerbestrbetae, Birkenfeld, Germany) Unit of Nuclear Medicine Laboratory of the hospital to rule out any endocrinal abnormality.

Trans-vaginal sonography (TVS) was done with a 7.5 *MHz* endo-vaginal probe (Color Doppler Ultrasound Machine Model Sonaace 8000Ex Prime Sr No 66505300001993). On the transverse view of uterus, uterine artery blood flow was measured using 2-D-PCD at the level of internal cervical os. The insonation angle was between 0° and 30°. The PI, RI, PSV, S/D were measured in left and right uterine arteries.

Based on the age along with AFC and baseline hormonal levels, these women were put on agonist or antagonist protocol.

On 2-D-PCD, the Resistance Index (RI=(PSV-EDV)/PSV=(peak systolic velocity-end diastolic velocity)/peak systolic velocity), the Pulsatility Index (*PI*=(peak systolic velocity-end diastolic velocity)/mean velocity=(PSV-EDV)/TAV), the systolic/diastolic ratio S/D (systolicvelocity/ diastolic velocity) were calculated on three consecutive wave forms which were used to evaluate uterine artery resistance.

### Statistical analysis:

Statistical analysis was performed by SPSS software (version 21.0; SPSS S.L., Madrid, Spain). Data were checked for normality before statistical analysis using Shaipro Wilk test. Data were presented as a number (%)/ mean±standard deviation (SD)/median (range). The differences in mean values were compared between pregnant and non-pregnant groups of women using Student’s t-test (normal data distribution)/Wilcoxon Ranksum Test (for categorical variables). The categorical variables were analyzed using the Chi-square test or Fisher’s exact test as appropriate. For all statistical tests, p<0.05 was considered to indicate a significant difference. All tests of statistical significance were two tailed.

## Results

A total of 188 women who underwent IVF/ICSI treatment were investigated. Among 188 women, 76 (40.4%) women became pregnant whereas 112 (59.60%) women did not conceive. These women were divided into two groups: Group A (the pregnant group; n=76) and Group B (the non-pregnant group; n=112). One hundred sixty nine patients had IVF cycles while 19 had ICSI cycles. Seventy one patients who underwent IVF and 5 patients who underwent ICSI conceived. The pregnancy rates were similar on day 3 and day 5 transfers with no significant difference (Table 1).

**Table 1.** Pregnancy rates on day 3 and day 5 transfer

**Day of transfer**	**No of patients**	**Positive**	**p-value**
**Day 3**	119	49 (41.1%)	0.877
**Day 5**	69	27 (39%)	

Both groups were also assessed on the basis of the duration of infertility. It was found that 69 (90.79%) and 96(85.71%) of women had ≤5 years of infertility in groups A and B, respectively while 7(9.21%) and 16(14.29%) of women had >5 years of infertility duration which were comparable.

The various etiological factors accounting for subfertility were assessed. It was observed that 79(42%) women had bilateral blocked tubes (tubal factor) and 39(20.7%) women were found to have lower sperm count according to WHO 2010 (male factor infertility). Unexplained infertility was seen in 38(20.2%) women and 10(5.3%) women were found with polycystic ovarian syndrome (PCOS), whereas endometriosis attributed in 12 (6.3%) women as a cause of their infertility. It was also observed that history of treatment for genital tuberculosis was noted in 9(11.84%) and 25(22.32%) patients in group A and group B respectively, while 28(36.84%) and 25(22.32%) had treatment for extra-genital tuberculosis. There were no significant differences between the pregnancy rates based on the ovarian stimulation protocols used (p=0.425). Also, the mean number of oocytes retrieved and embryos transferred amongst the patients in groups A and B were comparable (Table 2).

**Table 2.** The cycle characteristics in pregnant (Group A) and non-pregnant group (Group B)

**Parameters**	**Group A (Pregnant)**	**Group B (Non-pregnant)**	**p-value**
**Endometrial thickness on day of hCG trigger (*mm*)**	7.25±4.3	7.23±4.22	0.980
**Number of oocytes (n)**	9.75±5.92	9.32±6.29	0.677
**Number of embryos transferred (n)**	2.10±0.75	2.09±0.76	0.44

The 2D-PCD findings were sub-divided according to etiology and were compared consecutively during early follicular phase (on day2/3) and at the time of hCG trigger as shown in table 3. The PI measurements were found to be significantly raised in the early follicular phase in women with male factor infertility (p=0.0425*) and endometriosis (p=0.0196*) compared to those with other causes. The PI measured in unexplained infertility and known causes of infertility were compared and they found to be significant. The measured PI was high in known causes since they were in stress-hence-yoga therapy and counseling may be advocated for stress relief in women undergoing IVF treatment.

**Table 3.** Variation in uterine artery 2-D-PCD parameters based on different etiology

**Doppler Parameters**	**Male Factor**	**Other etiology**	**p-value**
**PSV (*cm/s*)**	24.26±7.09	27.98±6.57	0.0057^**^
**PI**	2.36±1.19	1.88±0.95	0.0425^*^
	**Endometriosis**	**Other causes**	--
**PI**	2.04±1.03	1.34±0.74	0.0196^*^
	**Unexplained factor**	**Known causes**	--
**PI**	1.85±0.56	2.12±0.64	0.0046^*^

The differences in the basal PI measurements in early follicular phase on day 2/3 of cycle were studied and it was found that before initiation of stimulation, the mean PI was 2.09±1.15 in group A and 1.9±0.95 in group B (p=0.385) which were comparable. Similarly, the mean PI measurements on the day of hCG injection were 1.86±1.12 in Group A, and 2.03±1 in Group B (p=0.192) and did not differ significantly (Table 4, 5).

**Table 4.** Comparison of basal uterine artery 2-D on day 2/3 of cycle in Pregnant and Non-Pregnant women

**Doppler Parameters**	**Group A (Pregnant)**	**Group B (Non-pregnant)**	**p-value**
**PSV**	26.23±6.95	27.87±6.68	0.127
**RI**	1.31±1.89	1.12±1.42	0.901
**PI**	2.09±1.15	1.9±0.95	0.385
**S/D**	13.11±17.27	9.74±13.81	0.520

**Table 5.** Comparison of uterine artery 2-D-PCD parameters at the time of hCG trigger in Pregnant and Non-Pregnant women

**Doppler Parameters**	**Group A (Pregnant)**	**Group B (Non-pregnant)**	**p-value**
**PSV**	26.17±8.94	24.63±7.41	0.126
**RI**	0.96±0.90	0.98±0.85	0.170
**PI**	1.86±1.12	2.03±1	0.192
**S/D**	11.14±17.48	18.92±30.81	0.081

hCG, human chorionic gonadotropin; PSV, peak systolic velocity; PI, pulsatility index; RI, Resistance Index, S/D systolic and diastolic ratio

The RI measurements were also studied between the two groups before starting the ovarian stimulation and on the day of hCG trigger. The differences in the mean basal RI measurements as well as the mean RI on the day of trigger were found non-significant between Group A and Group B (Table 4, 5). There were no significant changes between the mean uterine artery PSV and S/D during the cycle.

Using logistic regression analysis, the cutoffs for the various uterine artery doppler parameters were estimated which helped to predict successful conception. Thus, the power of the study was assessed that represented its accuracy and manifested the results more prominently. The cut off values for PI and RI were 2.05 and 0.9, respectively while for PSV and S/D, they were 27.75 and 2.05, respectively. As shown in table 6, the uterine artery resistance index (RI) <0.9 was the most sensitive predictor of successful pregnancy outcome and the S/D ratio seemed to be the least sensitive and predictive marker. This represents the power of the study.

**Table 6.** Sensitivity and specificity of doppler parameter in pregnant group of women

**Parameters**	**Associated criterion**	**Sensitivity**	**Specificity**	**PPV**	**NPV**
**PSV**	>27.75	73.91	58.93	52.5	78.5
**PI**	<2.05	75.36	41.96	44.4	73.4
**RI**	<0.9	92.75	22.32	75.36	83.3
**S/D**	<4.2	52.17	64.29	47.3	68.5

PPV, NPV: Positive predictive value, Negative predictive value

Although uterine artery 2D-PCD parameters in both group A and group B were not significantly different, the parameters of women in group B showed evidence of decreased uterine perfusion as compared to group A during hCG trigger.

## Discussion

The pregnancy rate in IVF-ET cycles depends on a wide array of factors associated with different aspects of assisted reproduction. Uterine blood flow parameters might be one of these factors affecting implantation. Steer et al. (1995) suggested that poor uterine perfusion determined by uterine artery color doppler could be a cause for implantation failure among unsuccessful IVF patients (3). This assessment prompted the present study where the quantitative measurement of PSV, RI, PI and S/D of uterine artery was conducted and the correlation between the same and the pregnancy rates was sought in ART cycles.

Cacciatore et al. (1996) observed that PI and RI were lower in conception (2.45±0.54 and 0.85± 0.04) as compared to non-conception cycles (2.66±0.39 and 0.87±0.04) (4). The overall pregnancy rate was 35% per embryo transfer and it decreased significantly when PI was >3.0 and RI>0.92 and only 13% of women got pregnant; when PI was >3.3 and RI>0.95, only 10% of women got pregnant. Such high impedance in fertility outcome was detected in 9% of IVF failed cycles.

On the contrary, other researchers have conducted similar studies but no significant difference was observed in PI and RI values in conception and non-conception cycles (11, 12). The present study too did not find any significant association between the basal and midcycle doppler parameters of women undergoing IVF and the pregnancy rates. It was also observed that there were no significant differences in doppler velocimetry measurements between conception and non-conception cycles including fresh and frozen embryo transfers (FET) cycles.

Optimum uterine receptivity seemed to occur when mean pulsatility index of both left and right uterine arteries were between 2 and 3 (13, 14). Pregnancy rate decreased significantly when pulsatility index was more than 3 or 4 (15, 16); however, diastolic flow was not observed in doppler wave form (17). In our study, the pregnant group of women had the mean PI of 2.09±1.15 and 1.86±1.12 during early follicular phase and on the day of hCG trigger, respectively.

Our findings also suggest that doppler parameters may vary in women with different etiologies of infertility. It was found that women with male and tubal factors have less PSV and uterine perfusions as compared to the women with unexplained infertility. Unpublished research at our centre has shown that couples who are aware that either one or both of them have a known pathology accounting for infertility, have higher stress levels leading to poorer psychological milieu compared to those with unexplained infertility.

De-stressing techniques like counseling and/or yoga which may help to reduce stress levels in women that can lead to higher uterine artery circulation is favorable for implantation. In addition, further investigations are required on the associations of different uterine factors having higher impacts on successful pregnancy outcome in women undergoing IVF cycles.

Good endometrium triple line pattern, oocyte quality, and numerous autocrine, paracrine and endocrine factors inexorably influence the embryo endometrium cross talk that assessment of low or high uterine artery perfusion alone fails to correlate significantly with implantation rates. Thus, uterine artery 2D-PCD indices may be used with some of these factors to form a predictive algorithm for IVF pregnancy rates but independently is not a predictor for IVF implantation or pregnancy rates.

## Conclusion

It was concluded that early and late follicular phase uterine artery doppler indices are not reliable predictors of IVF cycle pregnancy rates. Nevertheless, the uterine artery RI<0.9 most closely correlated with successful pregnancy outcome. An interesting observation was that uterine vascularity indices varied significantly between women with known etiology and unexplained infertility undergoing IVF. Women with known etiology contributing to infertility were noted to have higher impedance to uterine perfusion compared to those with unexplained infertility. This finding opens new portals to encourage further research especially encompassing the subliminal factors concerning infertility and its management.
